# Optical genome mapping refines cytogenetic diagnostics, prognostic stratification and provides new molecular insights in adult MDS/AML patients

**DOI:** 10.1038/s41408-022-00718-1

**Published:** 2022-09-02

**Authors:** Estelle Balducci, Sophie Kaltenbach, Patrick Villarese, Eugénie Duroyon, Loria Zalmai, Chloé Friedrich, Felipe Suarez, Ambroise Marcais, Didier Bouscary, Justine Decroocq, Rudy Birsen, Michaëla Fontenay, Marie Templé, Chantal Brouzes, Aurore Touzart, Thomas Steimlé, Agata Cieslak, Ludovic Lhermitte, Carole Almire, Nicolas Chapuis, Olivier Hermine, Vahid Asnafi, Olivier Kosmider, Lucile Couronné

**Affiliations:** 1 Laboratory of Onco-Hematology, Hôpital Necker Enfants-Malades, Assistance Publique-Hôpitaux de Paris (APHP), Paris, France; 2 INSERM U1151, Institut Necker Enfants Malades (INEM), Paris, France; 3 Laboratory of Hematology, Hôpital Cochin, Assistance Publique-Hôpitaux de Paris, Paris, France; 4 Hematology Department, Hôpital Necker Enfants-Malades, Assistance Publique-Hôpitaux de Paris (AP-HP), Paris, France; 5 Department of Clinical Hematology, Hôpital Cochin, Assistance Publique-Hôpitaux de Paris, Paris, France; 6 Laboratory of Cellular and Molecular Mechanisms of Hematological Disorders and Therapeutic Implications, INSERM U1163, Imagine Institute, Paris City University, Paris, France; 7 OPALE Carnot Institute, The Organization for Partnerships in Leukemia, Paris City University, Paris, France

**Keywords:** Cancer genetics, Genetics research

Dear Editor,

Myelodysplastic syndromes (MDS) and acute myeloid leukemia (AML) are clinically and genetically heterogeneous myeloid malignancies associated with a broad range of recurring mutations and cytogenetic abnormalities. To date, their diagnostic workup includes a conventional karyotype to establish the IPSS-R (Revised International Prognostic Scoring System) and ELN (European Leukemia Net) prognostic scores in MDS and AML patients, respectively [1–3].

In recent years, many advances have been made in the development of methods for the detection of somatic cytogenetic abnormalities. In this regard, optical genome mapping (OGM) is a cutting-edge technology developed for genome-wide detection of structural variants (SVs) including balanced and unbalanced translocations, inversions, insertions, deletions, duplications as well as copy number variations (CNVs). This technology is based on the comparative analysis of optical genome maps obtained from high molecular weight DNA greater than 150 kb. OGM aims to address the limitations of existing cytogenetic techniques offering a higher resolution than karyotyping, allowing whole-genome analysis, unlike FISH, and the detection of balanced chromosomal abnormalities missed by CGH/SNP-array analysis [4]. Recently, its assessment in hematological malignancies including myeloid neoplasms has shown promising results as compared with standard cytogenetic techniques [5–9].

Given the scarce data available on this new technology, we conducted a study on a French series of 68 adult MDS and AML patients to evaluate its performance in the detection of somatic cytogenetic abnormalities and its clinical utility.

Sixty-eight samples (64 bone marrow samples and 4 peripheral blood samples) including 27 MDS cases and 41 AML cases were analyzed using OGM (Table S1). Twenty-six were retrospective cases collected between March 2010 and August 2020 and 42 were prospective cases sent to our laboratory for routine cytogenetic analysis between January 2021 and December 2021 (Fig. S1). Routine cytogenetic results were normal in 12/27 (44%) MDS cases and 19/41 (46%) AML cases, simple abnormal (<3 abnormalities) in 9/27 (33%) MDS cases and 12/41 (29%) AML cases, and complex (≥3 abnormalities) in 5/27 (18%) MDS cases and 8/41 (19%) AML cases. In the three remaining cases (1 MDS case and 2 AML cases), the karyotype was a failure. Patients’ routine cytogenetic and OGM results are presented in (Table S2).

OGM successfully detected most of the cytogenetic abnorma-lities seen on routine cytogenetics including aneuploidies as Y loss (Fig. S2A–C), monosomy 7, trisomy 8 (Fig. S2D–F), hyperdiploidy, and unbalanced SVs as 7q deletion (Fig. S2G–I), 5q deletion, ring of chromosome 7, 11q deletion, 20q deletion (Fig. 1). OGM also detected balanced SVs identifying partner genes of driver genes not routinely sought by other techniques such as t(6;11)/*KMT2A::MLLT4* (UPN 19) (Fig. S3A–C), inv(3)/*MECOM::RPN1* (UPN 87) (Fig. S3D–F), and t(2;3)/*MECOM::BCL11A* (UPN 134) (Fig. S3G-I and Fig. 1). Importantly, OGM provided successful analysis in the 3 cases of karyotype failure. Of note, in 10 patients, OGM missed cytogenetic abnormalities seen on routine cytogenetics in the following cases (Table S3): (1) low subclonal CNVs involving a whole chromosome as Y loss and trisomy 8, (2) clone with a gain of a whole batch of chromosomes as tetraploidy and near triploidy, (3) low subclones, (4) SVs which breakpoints are located in poorly covered areas by OGM (e.g. centromeric and telomeric regions) such as additional material on the short arm of acrocentric chromosomes. In two cases with available material (UPNs 1 and 112), we performed an interphase FISH analysis, known to display a low detection threshold. In UPN 112, interphase FISH detected trisomy 13 in 1.5% of the interphasic nuclei, confirming that OGM is not efficient to detect low subclonal trisomies. In UPN 1, interphase FISH failed to detect trisomy 5, indicating that a selective advantage of mitotic cells carrying aneuploidies under culture conditions cannot be fully ruled out. Overall, cytogenetic abnormalities seen on routine cytogenetics were successfully detected by OGM in 85% (58/68) of patients.

**Fig. 1:**
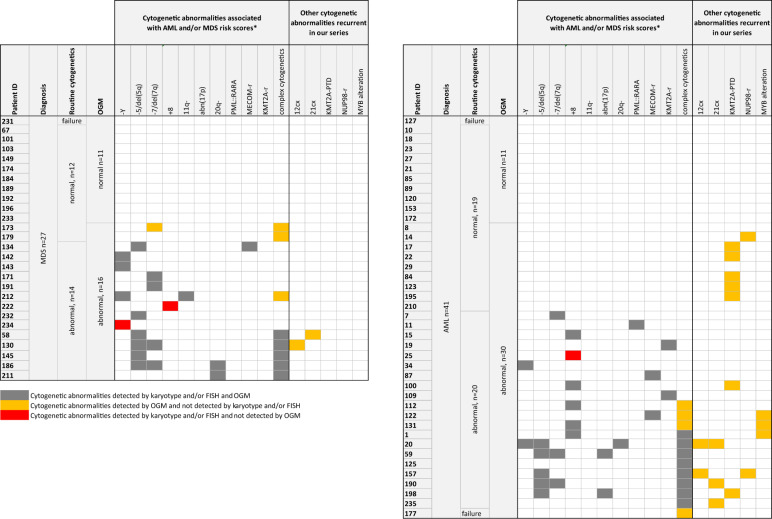
Summary of cytogenetic abnormalities detected in AML (left panel) and MDS (right panel) cases according to the cytogenetic method (karyotype/FISH versus OGM). Cytogenetic results reported here include the cytogenetic abnormalities associated with AML and/or MDS risk scores and the other cytogenetic abnormalities recurrent in our series. Concordant results as well as abnormalities only detected by one of the technique are indicated. *detected categories only; cx complex rearrangement; r rearrangement.

In other cases, OGM revealed unexpected findings in cytogenetic abnormalities presumed to be simple by routine techniques. For example, in UPN 122, the translocation interpreted as t(3;14) did involve two breakpoints on the long arm of chromosome 3 located in the *RPN1* and *MECOM* genes in addition to the breakpoint on chromosome 14 located in the *KTN1* gene (Fig. S4). Other examples are shown in Figs. S5 and S6.

OGM also enables the elucidation of cytogenetic abnormalities not recognizable by karyotype because of their complexity or the poor quality of the karyotype. For example, OGM was able to define the chromosomal origin of all additional materials not recognized at karyotype in UPN 1 (Fig. S7). In addition, OGM detected recurrent complex (“cx”) rearrangements involving chromosomes 12 and 21, including some considered as chromothripsis-like (“cth”) SVs (Fig. 1). Complex rearrangements involving chromosome 12 were observed in 3/13 MDS/AML patients with a complex karyotype (UPNs 20, 130, and 157) (Fig. 2A–C and Fig. S8). All were associated with a deletion of the *ETV6* gene. Interestingly, other types of *ETV6* alterations such as mutations, rearrangements or haploinsufficiency have been previously reported in myeloid malignancies [10]. Complex rearrangements involving chromosome 21 were detected in 4/13 MDS/AML patients with a complex karyotype (UPNs 20, 58, 190, and 235) (Fig. S9). Amplification of the *ERG* gene was observed in all these cases and was associated with an amplification of the *RUNX1* gene in 3/4 cases. Until now, this abnormality has probably been considered as an intrachromosomal amplification of chromosome 21 and underestimated in AML [11].

**Fig. 2:**
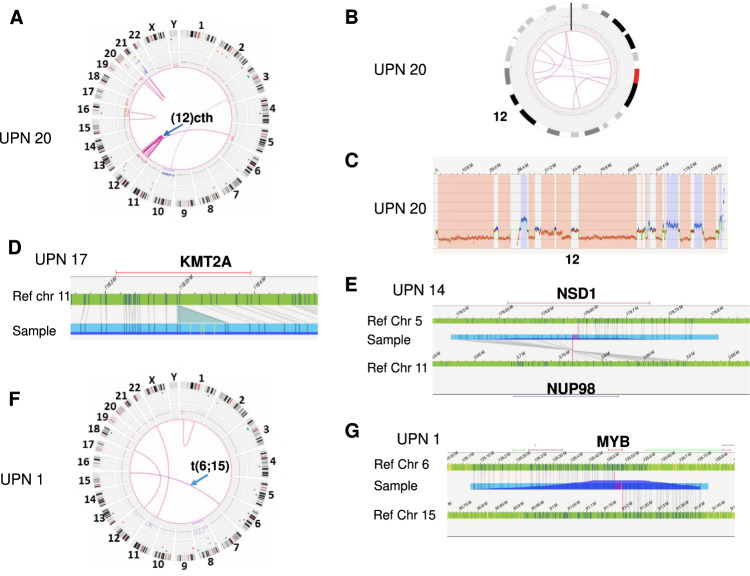
Examples of recurrent cytogenetic abnormalities detected by OGM in MDS/AML. **A**–**C** OGM results of UPN 20. **A** Circos plot revealing chromothripsis affecting the chromosome 12 (blue arrow). **B** Circos plot focusing on chromosome 12 showing multiple translocations (pink lines), gains (blue boxes), and losses (red boxes) affecting the chromosome 12. **C** CNV profile of the chromosome 12 showing multiple gains (blue boxes) and losses (red boxes). **D** Fine mapping of the long arm of chromosome 11 in UPN 17 indicating an insertion at 11q23.3 in *KMT2A* interpreted as *KMT2A* partial tandem duplication. **E** Fine mapping of the chromosomes 5 and 11 in UPN 14 indicating a translocation t(5;11)(q35.3;p15.4)(176625935;3755942) with a breakpoint located in *NSD1* on 5q35.3 and *NUP98* on 11p15.4. **F**, **G** OGM results of UPN 1. **F** Circos plot showing a balanced translocation between chromosomes 6 and 15 (blue arrow). **G** Fine mapping of the chromosomes 6 and 15 indicating a translocation t(6;15)(q23.3;q26.1)(135519170;91199438) with a breakpoint located in *MYB* on 6q23.3.

Overall, OGM detected additional relevant cytogenetic abnormalities in 17% (2/12) of MDS cases and 47% (9/19) of AML cases with normal routine cytogenetics, and in 22.2% (2/9) of MDS cases and 50% (6/12) of AML cases with simple abnormal routine cytogenetics (Table S2). In all cases with complex karyotypes (5 MDS and 8 AML cases) except UPN 125 with hyperdiploidy, OGM revealed an even more complex karyotype (Table S2). In AML cases, we identified three recurring classes of abnormalities (*i.e*. observed in at least two patients) namely partial tandem duplication of the *KMT2A* gene (*KMT2A*-PTD) (7/41 AML cases), alteration of the *MYB* gene (3/41 AML cases) and rearrangement of the *NUP98* gene (2/41 AML cases) (Fig. 1). In all cases, *KTM2A*-PTD was detected by OGM as an insertion in the *KMT2A* gene given the small size of the duplication (Fig. 2D and Fig. S10). *KMT2A*-PTD has been recently reported to be associated with an adverse prognosis in MDS patients in the novel molecular IPSS risk score, showing its importance [12]. Interestingly, *NUP98* rearrangements led to the formation of the *NUP98::NSD1* fusion gene in UPN 14 (Fig. 2E) and *NUP98::TNRC18* fusion gene in UPN 157 (Fig. S11). *NUP98* rearrangements are routinely investigated in childhood AML but to date, not in adult AML [13]. Alteration of the *MYB* gene consisted of duplication in UPN 122 (Fig. S12A, B), a translocation t(6;15) in UPN 1 (Fig. 2F, G) and an insertion ins(6;7) in UPN 131 (Fig. S12C, D). *MYB* overexpression has been described in AML, ALL, and lymphomas, and recurrent translocations and duplications of the *MYB* locus are known in T-ALL [14]. To our knowledge, only two case reports of SVs involving *MYB* have been reported in AML [14]. The recurrence of *MYB* alterations in our cohort supports that *MYB* might be a novel gene of interest in AML.

Lastly, we calculated the R-IPSS score or 2010/2017 ELN score and compared the scores obtained using cytogenetic data either by routine cytogenetics or by OGM. OGM is consistent with routine cytogenetics in 21/27 MDS and 39/41 AML patients regarding prognostic scores (Tables S4–S5). The R-IPSS risk score turned from favorable or intermediate to poor or very poor after OGM (UPNs 173, 179, 211 and 212) due to cryptic additional cytogenetic abnormalities affecting relevant genes such as *CUX1, SETD2, TET2*, and *PTPRT* leading to complex cytogenetics. The R-IPSS risk score turned from intermediate to favorable and very favorable to favorable in UPNs 222 and 234 respectively due to the non-detection of cytogenetic abnormalities such as trisomy 8 and Y loss. In UPN 109, the ELN risk score turned from adverse to intermediate prognosis because of the detection of a translocation t(9;11). In UPN 195 displaying normal karyotype and *NPM1* mutation, the identification of two additional cytogenetic abnormalities by OGM turned the prognosis from favorable to intermediate.

In summary, OGM resulted in a more complete assessment of complex cytogenetic events refining the underlying genomic structure reported by traditional cytogenetic methods and detected additional clinically relevant variants as well as potential novel candidate genes. In our series of 68 MDS/AML, OGM revealed the presence of cytogenetic abnormalities not seen at routine cytogenetics in 33% (9/27) and 54% (22/41) of the MDS and AML respectively. Thus, new prognostic scores integrating OGM and mutational data need to be designed in the future. Moreover, recommendations to interpret OGM data have to be established by an international expert panel and new rules have to be determined to redefine the ISCN nomenclature and the notion of cytogenetic complexity in the OGM era.

OGM is a very promising technology that has demonstrated its potential in the cytogenetic diagnostic workup of MDS/AML and opens the way for the identification of novel key players in myeloid pathogenesis.

## Supplementary information

Supplemental data

Table S1. Patients’ characteristics

Table S2. Patients’ routine cytogenetic and OGM results.

Table S3. Abnormalities missed by OGM analysis

Table S4. Prediction of risk score in MDS patients using IPSS-R risk score according to the cytogenetic method (karyotype/FISH versus OGM).

Table S5. Prediction of risk score in AML patients using ELN 2010/2017 risk scores according to the cytogenetic method (karyotype/FISH versus OGM).

Table S6. Technical performances of OGM analysis

Table S7. List of relevant genes involved in malignant hematological diseases

Table S8. Mutations in AML patients

## Data Availability

The datasets generated during and/or analysed during the current study are available from the corresponding author on reasonable request.
