# Supplementary material for: Optical genome mapping refines cytogenetic diagnostics, prognostic stratification and provides new molecular insights in adult MDS/AML patients
Source: Blood Cancer J. 2022 Sep 2;12(9):126. doi: 10.1038/s41408-022-00718-1 (PMC9440217; doi:10.1038/s41408-022-00718-1)
Supplement: Supplementary file 1 — Supplemental data [file 41408_2022_718_MOESM1_ESM.docx]

**SUPPLEMENTAL DATA**

**Optical genome mapping refines cytogenetic diagnostics, prognostic stratification and provides new molecular insights in adult MDS/AML patients**

**SUPPLEMENTAL METHODS**

**Sample selection**

Bone marrow (BM) aspirates or peripheral blood (PB) samples were obtained from MDS and AML adult patients from Necker and Cochin Hospitals after informed consent. All cases had been investigated by karyotyping (supplemented if necessary by FISH analyses). Patients were selected based on their cytogenetic profile to include a roughly equivalent number of patients with normal or abnormal karyotype in each entity.

A total of 87 samples were processed for OGM analysis. In 19 cases, DNA extraction failed. Overall, sixty-eight cases with successful OGM analysis were eventually included in this study. Twenty-six retrospective cases were collected between March 2010 and August 2020, and frozen in 10% DMSO after isolation of mononuclear cells using density-gradient separation (Ficol-Paque, GE Healthcare Life Sciences, Pittsburgh, PA) (Fig. S1). Forty-two prospective cases were sent to our laboratory for routine cytogenetic analysis between January 2021 and December 2021. In case of sufficient leftover material (*i.e*, more than 5 million cells after completion of routine cytogenetic tests), these samples were either extracted fresh or frozen at -80 °C after the addition of DNA stabilization buffer (Bionano Genomics San Diego, CA, USA) for OGM analysis in real-life.

**DNA extraction**

For each sample, 1.5 million cells from BM aspirates or PB samples were used to purify ultra-high molecular weight DNA using the Bionano Prep SP BMA DNA Isolation kit following manufacturer instructions (Bionano Genomics). Briefly, after counting, white blood cells were pelleted (16 000g for 2mn) and treated with lysis and binding buffer (LBB) and proteinase K to release genomic DNA (gDNA). After inactivation of proteinase K by PMSF treatment, gDNA was bound to a paramagnetic disk, washed, and eluted in an appropriate buffer. Ultra-High molecular weight DNA was left to homogenize at room temperature for two to three days before DNA quantitation using Qubit dsDNA assay BR kit with a Qubit 3.0 fluorometer (Thermo Fisher Scientific, Waltham, MA, USA).

**DNA Labeling**

DNA molecules were labeled using the Direct Label and Stain (DLS) DNA Labeling Kit (Bionano Genomics). Seven hundred and fifty nanograms of gDNA were labeled in presence of Direct Label Enzyme (DLE-1) and DL-green fluorophores. After clean-up of the excess of DL-Green fluorophores and rapid digestion of the remaining DLE-1 enzyme by proteinase K, the DNA backbone was counterstained overnight before DNA quantitation using Qubit dsDNA assay HS kit with a Qubit 3.0 fluorometer (Thermo Fisher Scientific).

**Chip loading and data collection**

A volume of 8.5µl of labeled gDNA solution of concentration between 4 and 12ng/µl was loaded on the Saphyr chip and scanned on the Saphyr instrument (Bionano Genomics). A maximum of three samples is loaded on a chip. Saphyr chips were run at maximum capacity to reach a minimum yield of 1500 Gbp.

**Data analysis**

All data were analyzed using the RVP from Bionano Genomics, which is dedicated to the detection of somatic cytogenetic abnormalities in cancer cells. The RVP was executed on Bionano Solve software V3.5. Direct visualization of SVs and CNVs was done on Bionano Access V1.6. OGM results are displayed on a CIRCOS plot showing a whole-genome view. The outer layer represents the G-bands of the chromosomes. The middle layer indicates the locations of the SVs identified in each chromosome. The inner layer represents the copy number profiles showing losses and gains.

**OGM quality data**

OGM of the 68 samples generated an average of 1 589 +/- 444 Gbp of data per sample with an average N50 molecule length (>150Kbp) of 0.300 +/- 0.055 Mbp, N50 molecule length (>20Kbp) of 0.235+/-0.072 Mbp, and label density of 15.7+/-1.5 labels/100Kbp (Table S6). The average map rate was 82.5+/-11.5% and the average effective coverage was 386+/-100X. 12/68 samples displayed an N50 molecule length (>150Kbp) lower than 0.230 Mbp and/or an N50 molecule length (>20Kbp) lower than 0.150 Mbp consisting of fragmented DNA samples. For 6 of these cases, the expected SVs were detected by the SV tool while the background noise made the interpretation of the CNV tool impossible.

**Data interpretation**

To identify high confidence relevant variants per sample, various filters were applied (Fig. S13). Variants were prefiltered based on Bionano Genomics recommended criteria: 1) Confidence scores: insertion, 0; deletion, 0; inversion, 0.7; duplication, -1; intra-translocation, 0.3; inter-translocation, 0.65, and copy number, 0.99 (low stringency, filter set to 0), 2) the size cutoff was set at 5Kb for insertions/deletions detected by the SV tool and 500Kbp for the CNV tool, 3) CNV fractional analysis was set to be lower than 1.8 for deletions or greater than 2.2 for duplications. To exclude polymorphic or artefactual variants, we filtered out the variants with the following characteristics: 1) supported by less than 5 self-molecules, 2) detected in healthy individuals by comparison to the Bionano Genomics database of 200 human control samples and the Database of Genomic Variants (DGV), 3) overlapping with difficult-to-map regions by comparison to the Bionano Genomics database of masked genomic regions, 4) corresponding with translocations displaying incorrect mapping or close to difficult-to-map regions. Among the remaining variants, we finally retained relevant SVs and CNVs as follows: 1) all CNVs with size > 500Kb, 2) all translocations not considered as artefactual as above-mentioned, and 3) all SVs regardless of their size if they overlap one of the genes defined as relevant in malignant hematological diseases (Table S7). This gene list was created by combining the leukemia-specific gene lists provided by the cancer genomics consortium (www.cancergenomics.org) and by Bionano Genomics. Variants smaller than 500Kb (i.e only detected by the SV tool) and not overlapping with a gene listed here were not addressed in the current study. Complex cytogenetics in OGM was defined by the presence of 3 or more cytogenetic abnormalities by analogy to the karyotype.

**Karyotyping**

Karyotypes with R-banding were performed on BM aspirates or PB samples using standard procedures. Whenever possible, at least 20 metaphases were analyzed, and the karyotype was described according to the International System for Human Cytogenetic Nomenclature (ISCN, 2020)^1^.

**Fluorescence in situ hybridization**

FISH was performed on cytogenetic preparations using commercial probes. At least 200 interphase cells were counted, and hybridization patterns were recorded. All available metaphases were analyzed. As recommended, in all cases of AML with normal karyotype, FISH covering *KMT2A* and *MECOM* genes were systematically carried out to detect specific rearrangements. When needed, FISH analysis was performed to confirm variants detected with OGM. The following probes from Metasystems (Altlussheim GmbH, Germany) were used according to the manufacturer's instructions: XL *KMT2A* break-apart probe, XL *MECOM* break-apart probe, XL 5q31/5q33 deletion probe, XL *PML/RARA* Translocation/Dual fusion probe, XL *DLEU/LAMP* deletion probe.

**Molecular analysis**

For AML patients, mutations included in the definition of the 2010 and 2017 ELN risk scores were screened by high throughput sequencing, fragment analysis approach or by Sanger sequencing as previously reported^2^. Results are shown in Table S8.

**OGM nomenclature**

We modeled a nomenclature adapted from the article by Levy et al. and the ISCN 2020 logic for karyotype, FISH, and microarray^1,3^.

**REFERENCES**

1 *ISCN 2020 | Karger Book*. https://www.karger.com/Book/Home/279152 (accessed 1 Feb2022).

2 Vazquez R, Breal C, Zalmai L, Friedrich C, Almire C, Contejean A *et al.* Venetoclax combination therapy induces deep AML remission with eradication of leukemic stem cells and remodeling of clonal haematopoiesis. *Blood Cancer J* 2021; **11**: 62.

3 Levy B, Baughn LB, Chartrand S, LaBarge B, Claxton D, Lennon A *et al.* A National Multicenter Evaluation of the Clinical Utility of Optical Genome Mapping for Assessment of Genomic Aberrations in Acute Myeloid Leukemia. 2020; : 2020.11.07.20227728.

**SUPPLEMENTAL FIGURES**

**
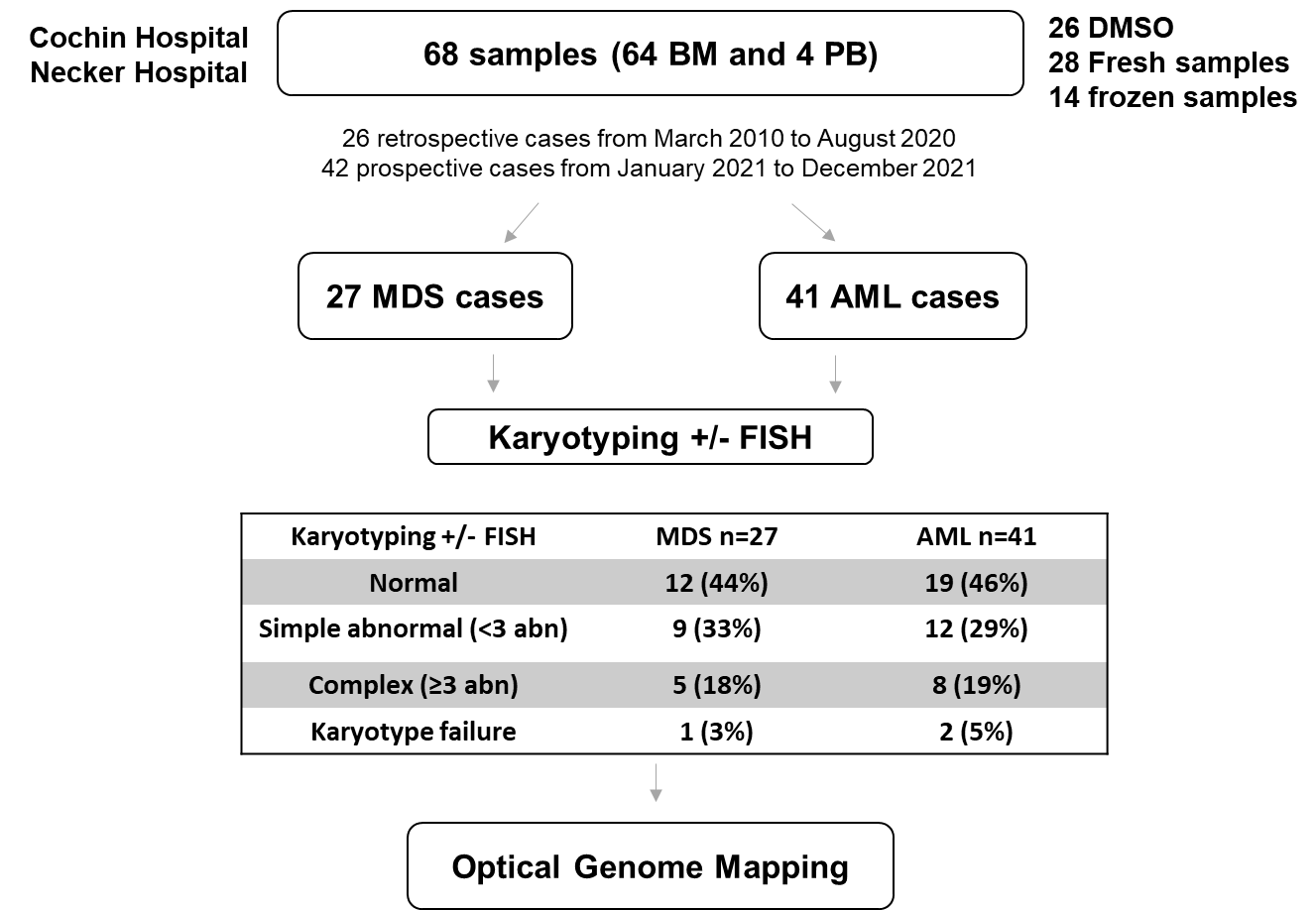
**

**Fig.S1. Design of the study**

BM, bone marrow samples; PB, peripheral blood samples; Abn, abnormalities;

**
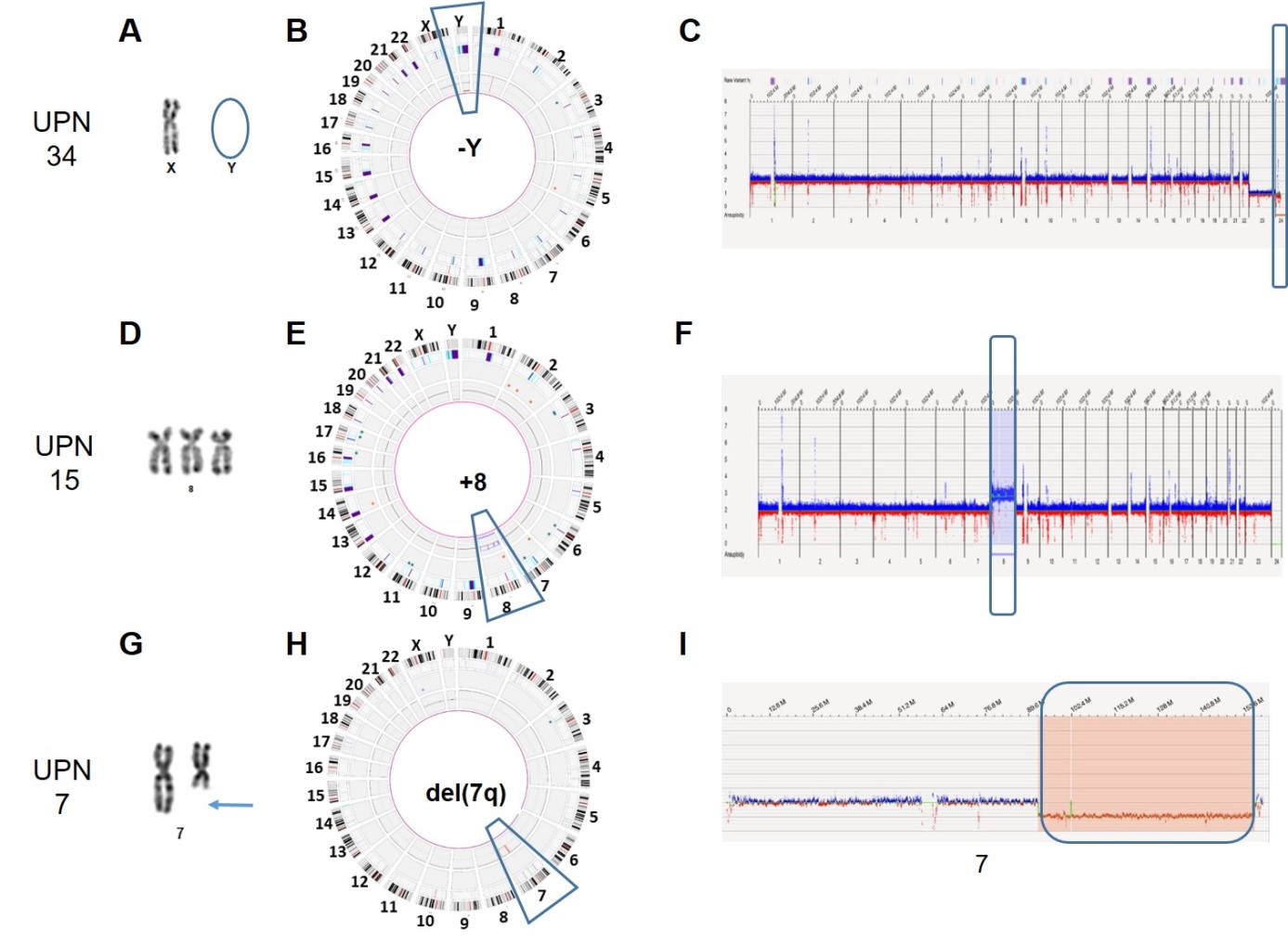
**

**Fig.S2. Representative examples of CNVs detected by OGM (UPNs 34, 15 and 7)**.

A-C) UPN 34. A) Partial R-banded karyotype showing loss of chromosome Y (blue circle). B) Circos plot showing a loss of chromosome Y (blue box). C) Whole-genome copy number profile showing loss of chromosome Y (blue box). D-F) UPN 15. D) Partial R-banded karyotype showing trisomy 8. E) Circos plot showing trisomy 8 (blue box). F) Whole-genome copy number profile showing trisomy 8 (blue box). G-I) UPN 7. G) Partial R-banded karyotype showing deletion of the long arm of chromosome 7 (blue box). H) Circos plot showing a deletion of the long arm of chromosome 7 (blue box). I) CNV profile of the long arm of chromosome 7 indicating a del(7)(q21.2q36.3)(92405783_156739761) sizing 64 Mb.

**
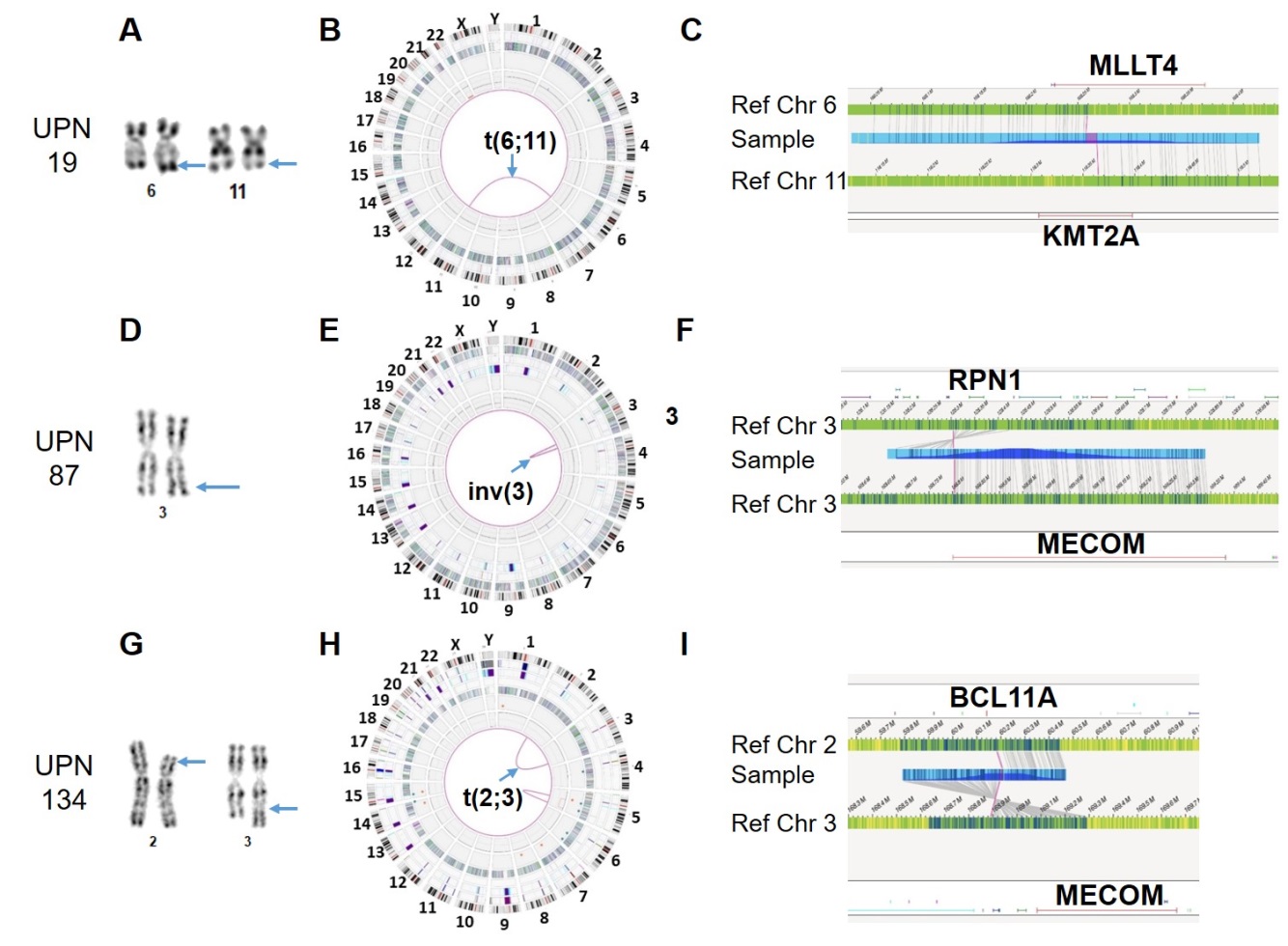
**

**Fig.S3. Representative examples of balanced translocation and inversion detected by OGM.**

A- C) UPN 19. A) Partial R-banded karyotype showing t(6;11)(q27;q23) (blue arrows). B) Circos plot showing a balanced translocation between chromosomes 6 and 11 (blue arrow). C) Fine mapping of the chromosomes 6 and 11 indicating a translocation t(6;11)(q27;q23.3)(168259152;118364657) with breakpoints located in *MLLT4* at 6q27 and *KMT2A* at 11q23.3 leading to a putative *MLLT4::KMT2A* gene fusion. D-F) UPN 87. D) Partial R-banded karyotype showing inv(3)(q21q26) (blue arrow). E) Circos plot showing a balanced paracentric inversion on the long arm of chromosome 3 (blue arrow). F) Fine mapping of the long arm of chromosome 3 indicating an inv(3)(q21.3q26.2)(128304581;168804024) with breakpoints located in *MECOM* on 3q26.2 and upstream of the *RPN1* gene on 3q21.3. G-I) UPN 134. G) Partial R-banded karyotype showing t(2;3)(p16.1;q26.2)(60178832;168882938) (blue arrows). H) Circos plot showing a balanced translocation between chromosomes 2 and 3 (blue arrow). I) Fine mapping of the chromosomes 2 and 3 indicating a translocation t(2;3)(p16.1;q26.2)(60178832;168882938) with breakpoints located in *BLC11A* on 2p16.1 and *MECOM* on 3q26.2.


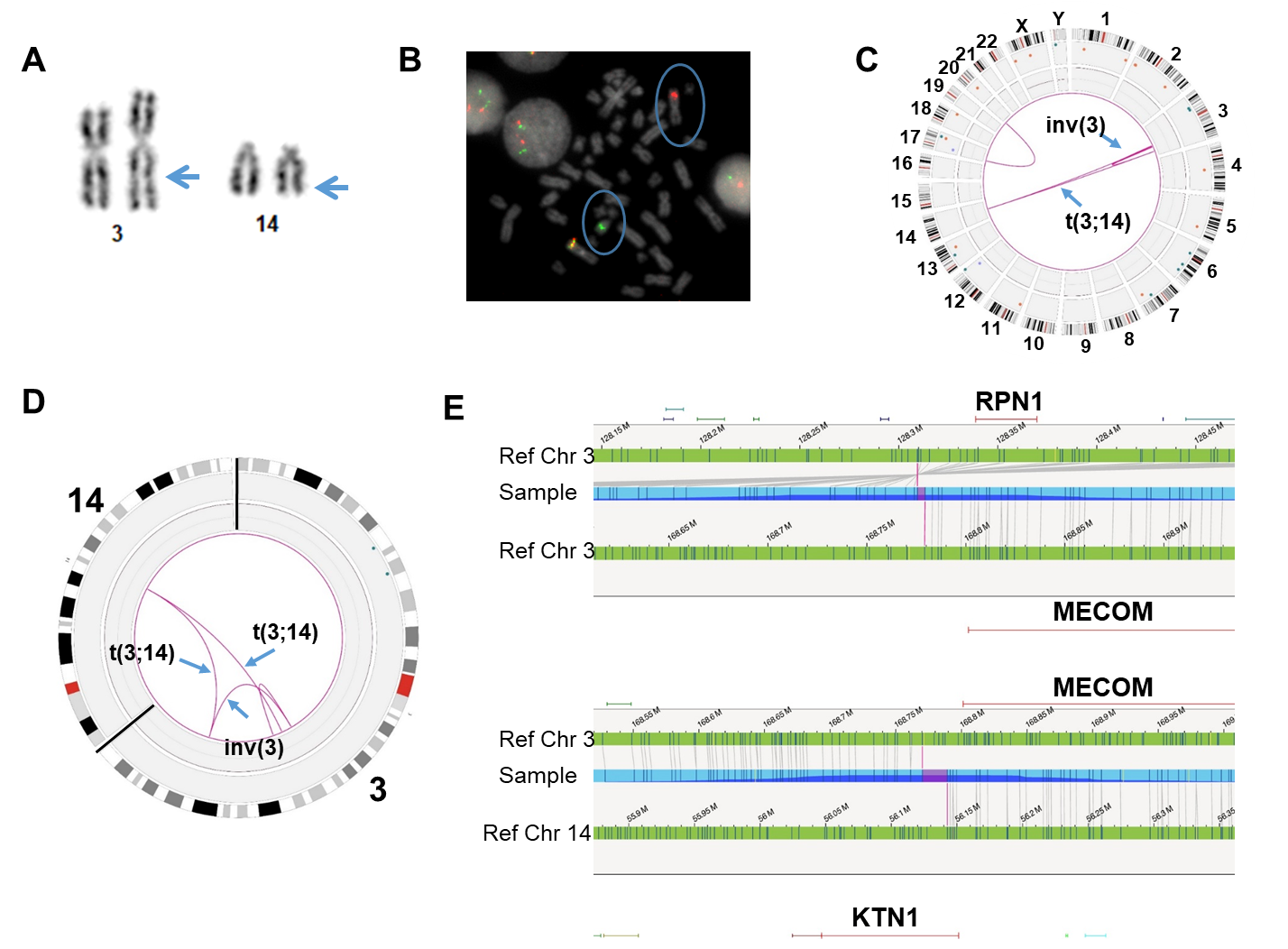


**Fig.S4. Example of complex SV affecting *MECOM* gene revealed by OGM (UPN 122)**

1. Partial R-banded karyotype interpreted as translocation t(3;14)(q26;q3?2). B) Metaphase FISH analysis showing *MECOM* rearrangement (blue circle) using XL *MECOM* break apart probe (Metasystems). C) Circos plot revealing two balanced translocations involving chromosomes 3 and 14 and an additionnal inversion of the long arm of chromosome 3 (blue arrows). D) Circos plot focusing on chromosomes 3 and 14. E) Fine mapping of chromosomes 3 and 14 showing an inversion inv(3)(q21.3;q26.2)(128309402;168779164) with breakpoints located in *RPN1* on 3q21.3 and *MECOM* on 3q26.2 and a translocation t(3;14)(26.2;q22.3)(168770278;56142693) with breakpoints located in *MECOM* on 3q26.2 and *KTN1* on 14q22.3.


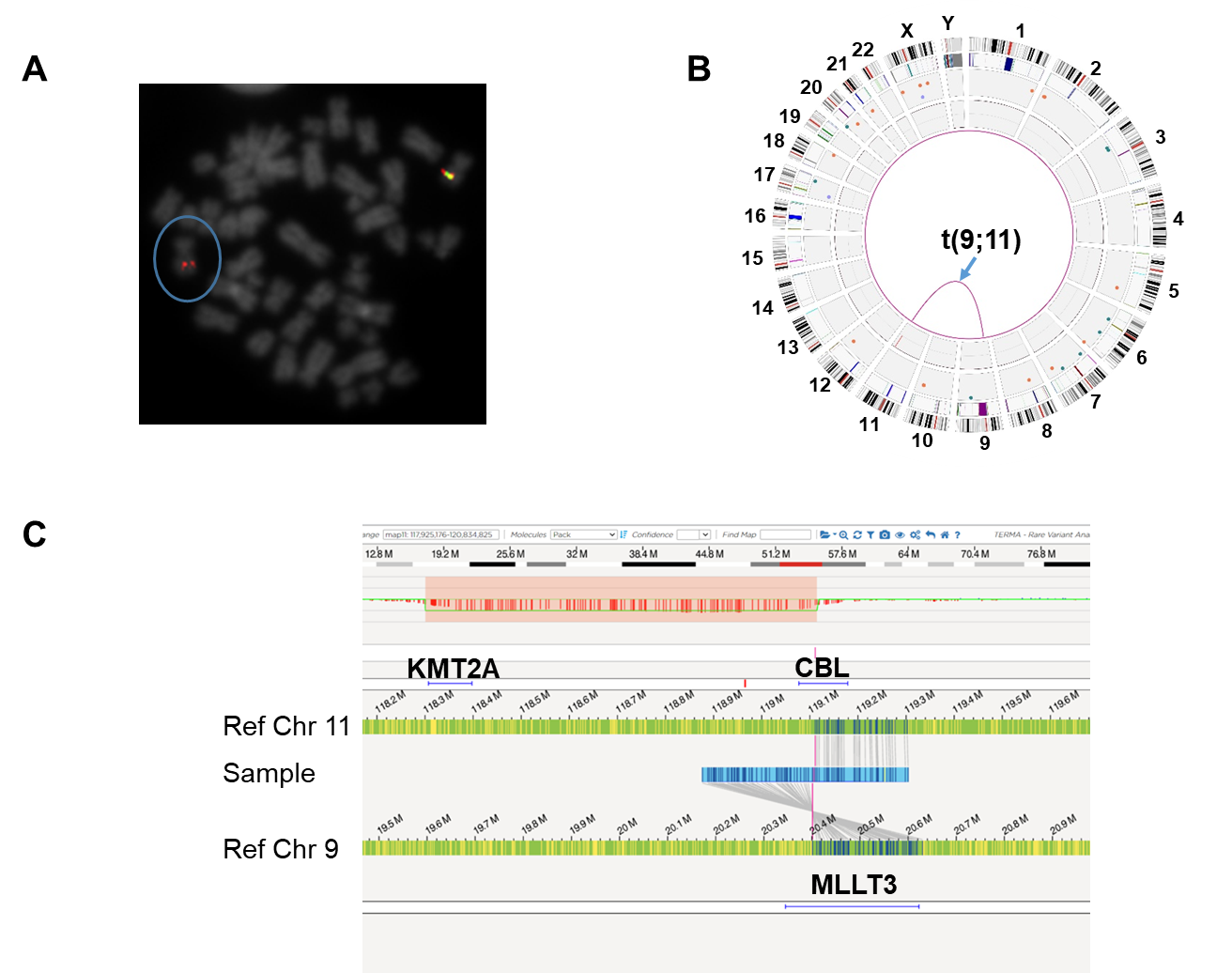


**Fig.S5. Example of complex SV affecting *KMT2A* gene revealed by OGM (UPN 109)**

A) Metaphase FISH analysis showing the deletion of the 3’ part of the *KMT2A* gene (blue circle) using XL *KMT2A* break apart probe (Metasystems). B) Circos plot revealing an unbalanced translocation involving chromosomes 9 and 11 (blue arrow). C) Fine mapping of the chromosomes 9 and 11 showing a translocation t(9;11)(p21.3;q23.3)(20401214;119111219) with breakpoints located in *MLLT3* on 9 p21.3 and *CBL* on 11q23.3. This translocation is associated with a deletion on 11q23.3 sizing 813Kb between the *KMT2A* and *CBL* genes leading to a putative *KMT2A::MLLT3*  gene fusion.

**
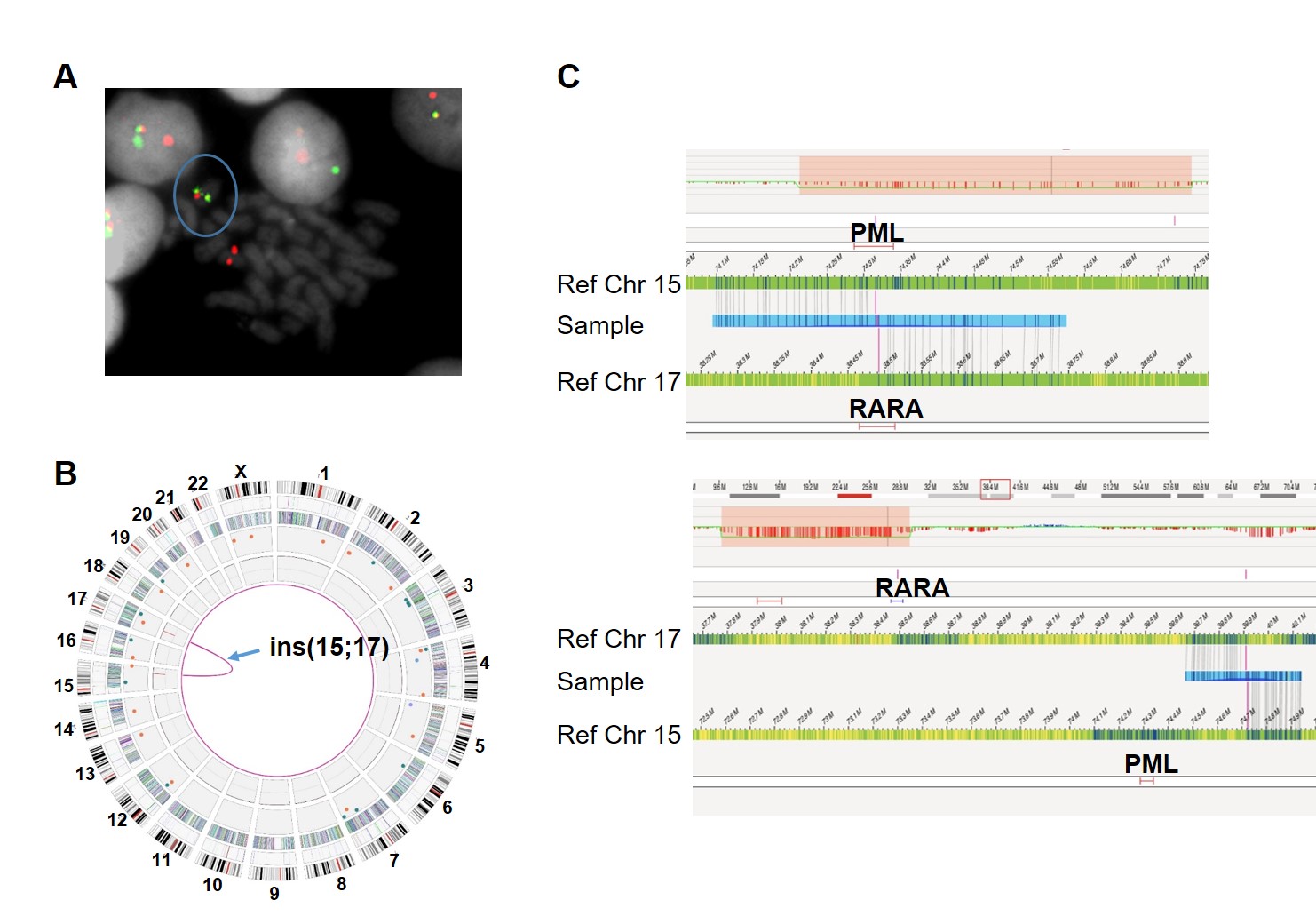
**

**Fig.S6. Example of complex SV affecting *PML* and *RARA* revealed by OGM (UPN 11)**

A) Metaphase FISH analysis showing the insertion of *RARA* in *PML* (blue circle) using FAST *PML::RARA* double fusion probe (Cytocell). B) Circos plot revealing an unbalanced translocation involving chromosomes 15 and 17 (blue arrow). C) Fine mapping of the chromosomes 15 and 17 showing an insertion ins(15;17)(q24.1;q21.2)(74316097;38491968_39908638) with breakpoints located in *PML* on 15q24.1 and *RARA* on 17q21.2 leading to a putative *PML::RARA* gene fusion. This translocation is associated with deletions at the breakpoints sizing 406Kb at 15q24.1 and 652Kb at 17q12-q21.2.

**
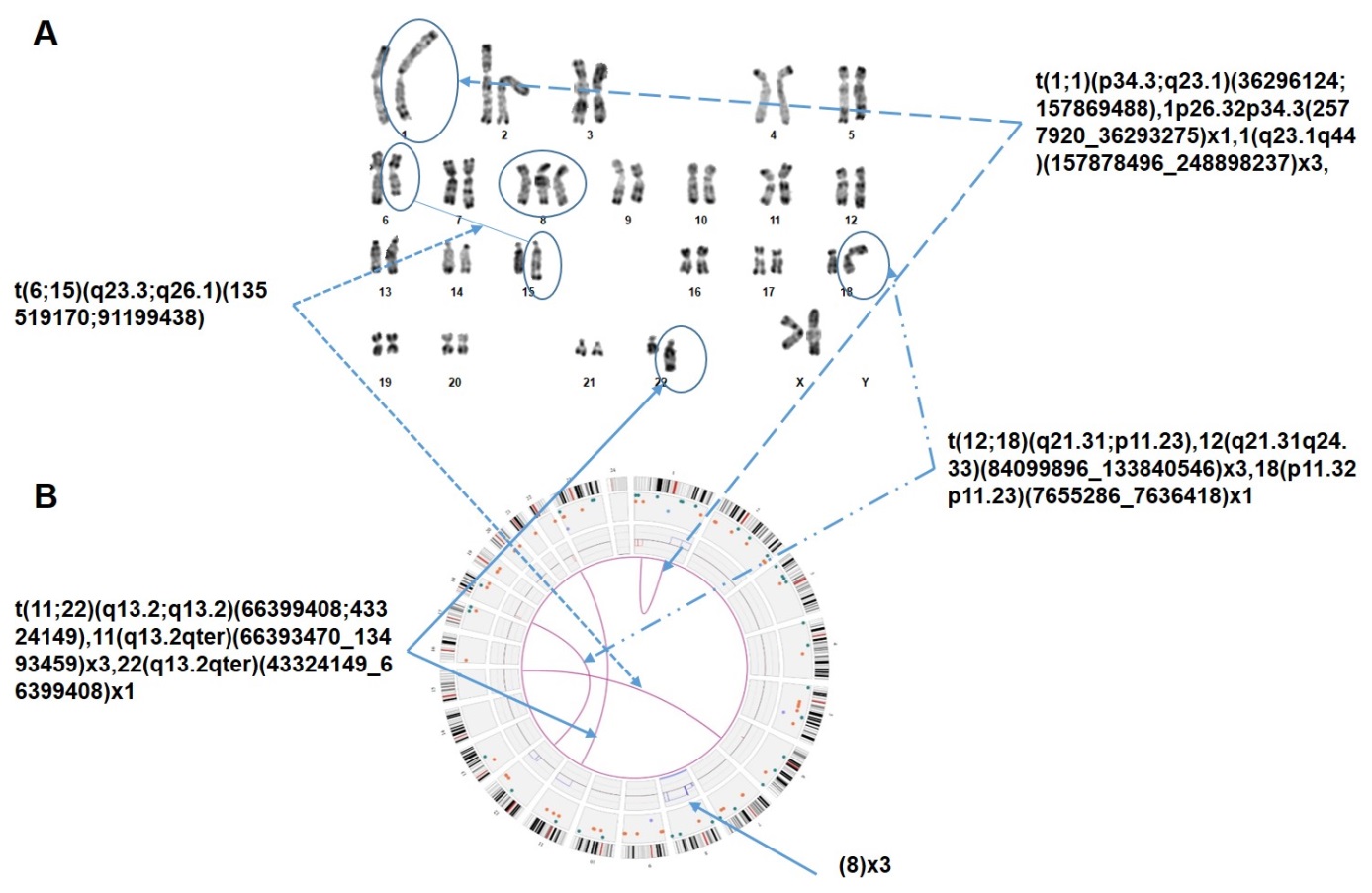
**

**Fig.S7. Example of complex karyotype analyzed by OGM (UPN 1)**

1. R-banded karyotype interpreted as

47,XX,add(1)(p31),del(6)(q14q22),+8,add(15)(q?),add(18)(p11),add(22)(q21)[12]. B) Optical mapping circos plot succeeded to identify all the additional materials not recognizable by karyotype. Karyotype was reinterpreted as 46,XY,der(1)t(1;1)(p34.3;q23.1),t(6;15)(q23.3;q26.1),+8,der(18)t(12;18)(q21.31;p11.23), der(22)t(11;22)(q13.2;q13.2).

**
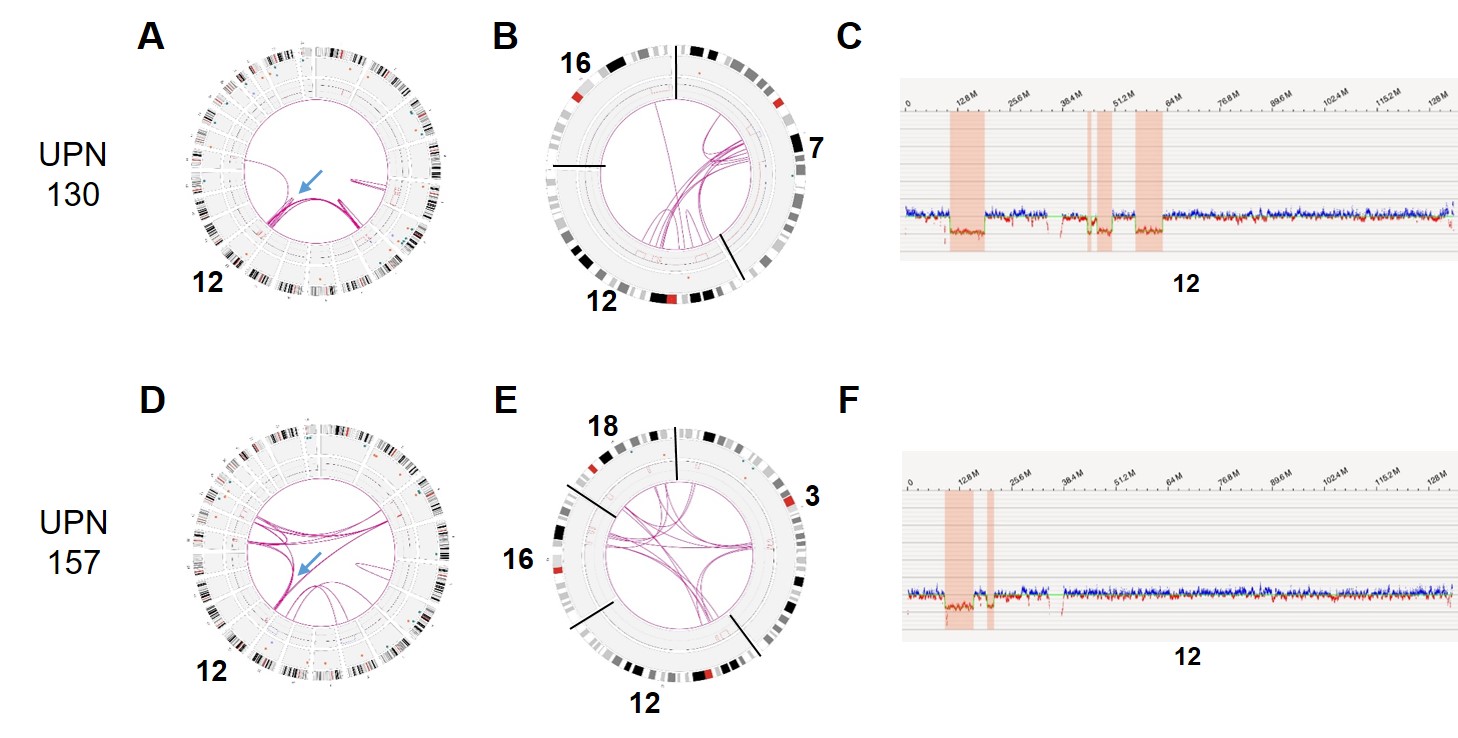
**

**Fig.S8. Examples of complex rearrangements involving chromosome 12 revealed by OGM (UPNs 130 and 157)**

A-C) UPN 130. A) Circos plot revealing complex rearrangements affecting the chromosome 12 but also the chromosomes 7 and 16 (blue arrow). B) Circos plot focusing on chromosomes 7, 12 and 16 showing multiple translocations (pink lines), gains (blue boxes) and losses (red boxes). C) CNV profile of the chromosome 12 showing losses (red boxes). D-F) UPN 157. D) Circos plot revealing complex rearrangements affecting the chromosome 12 but also the chromosomes 3, 16 and 18 (blue arrow). E) Circos plot focusing on chromosomes 3, 12, 16 and 18 showing multiple translocations (pink lines), gains (blue boxes) and losses (red boxes). F) CNV profile of the chromosome 12 showing losses (red boxes).

**
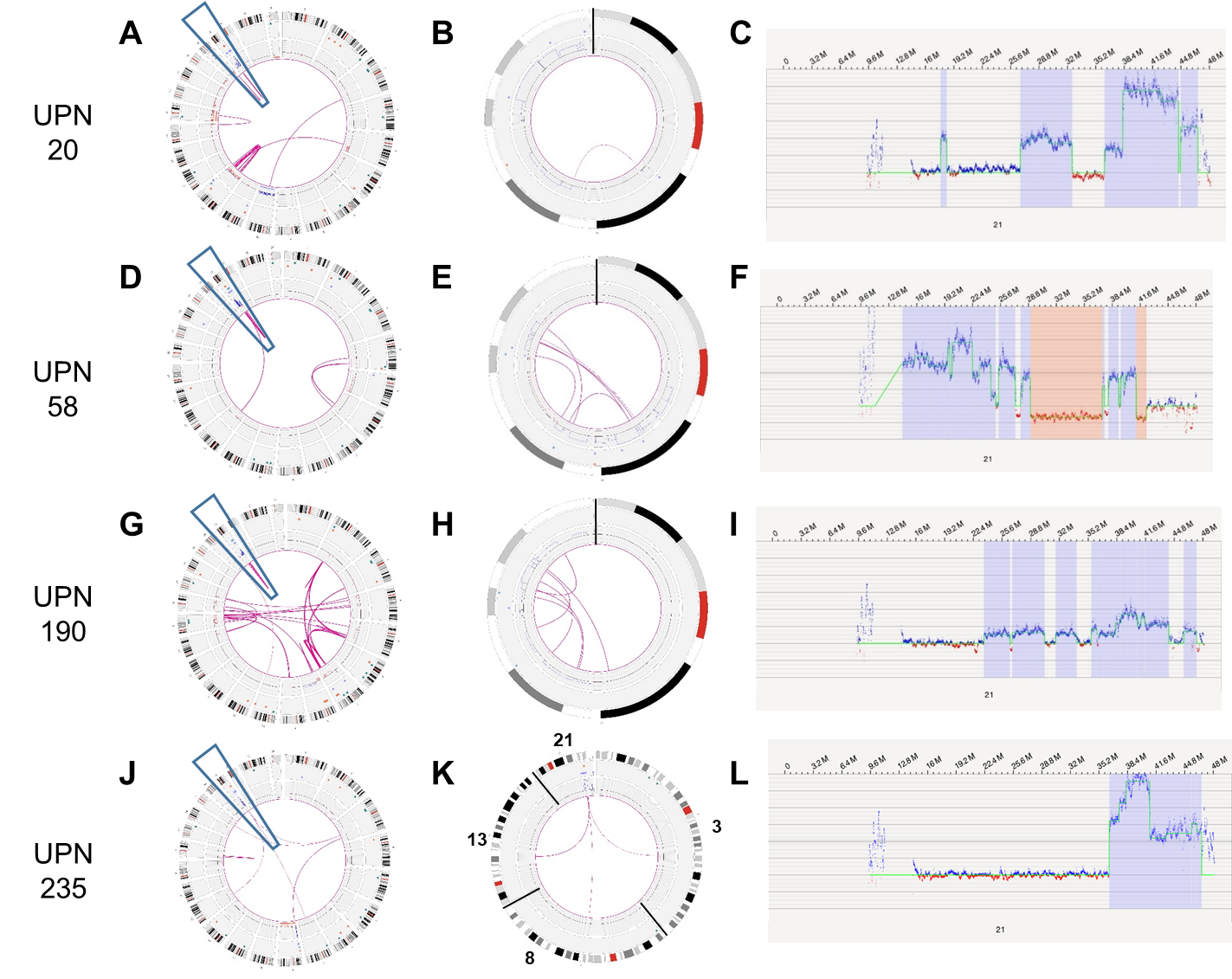
**

**Fig.S9. Examples of complex rearrangements involving chromosome 21 revealed by OGM (UPNs 20, 58, 190, and 235)**

A-C) UPN 20. A) Circos plot revealing complex rearrangements affecting the chromosome 21 (blue box). B) Circos plot focusing on chromosome 21 showing a translocation (pink line) and gains (blue boxes) affecting the chromosome 21. C) CNV profile of the chromosome 21 showing multiple gains (blue boxes). D-F) UPN 58. D) Circos plot revealing chromothripsis affecting the chromosome 21 (blue box). E) Circos plot focusing on chromosome 21 showing multiple translocations (pink lines), gains (blue boxes), and losses (red boxes). F) CNV profile of the chromosome 21 showing gains (blue boxes) and losses (red boxes). G-I) UPN 190. G) Circos plot revealing complex rearrangements affecting the chromosome 21 (blue box). H) Circos plot focusing on chromosome 21 showing multiple translocations (pink lines) and gains (blue boxes). I) CNV profile of the chromosome 21 showing gains (blue boxes). J-L) UPN 235. J) Circos plot revealing complex rearrangements affecting the chromosome 21 (blue box). K) Circos plot focusing on chromosomes 3, 8, 13 and 21 showing multiple translocations (pink lines) and gains (blue boxes). L) CNV profile of the chromosome 21 showing gains (blue boxes).

**
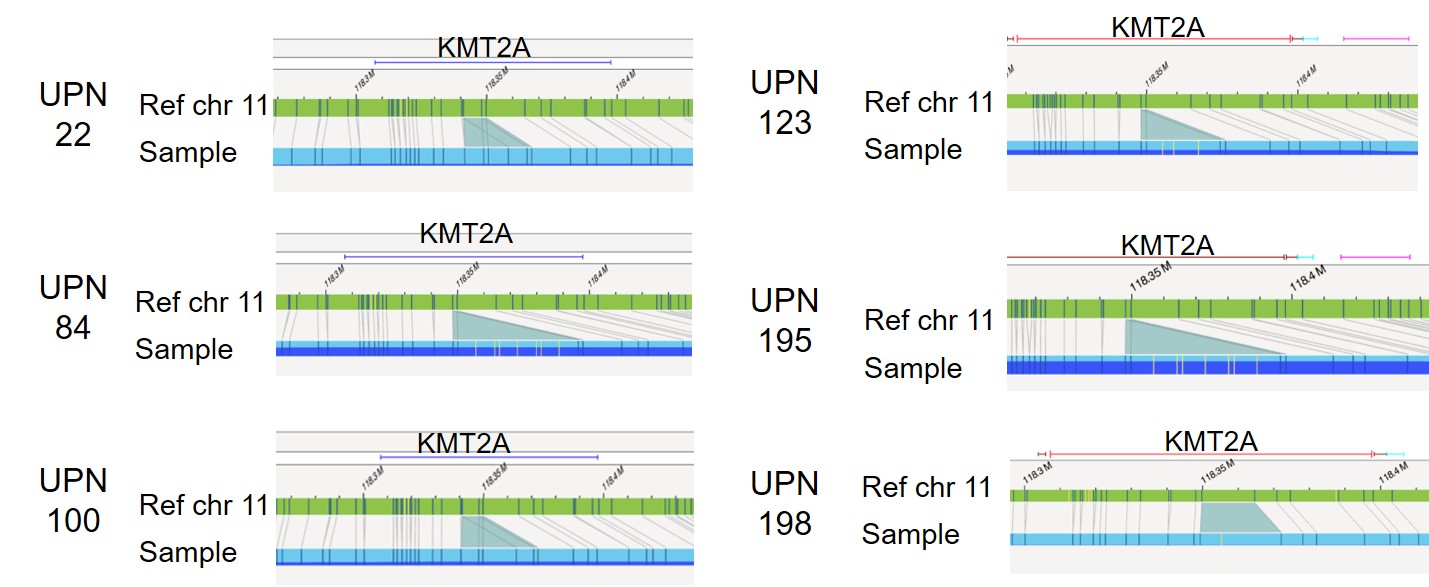
**

**Fig.S10. Examples of KMT2A partial tandem duplications revealed by OGM (UPNs 22, 84, 100, 123, 195, and 198)**

Fine mapping of the long arm of chromosome 11 indicating an insertion at 11q23.3 in *KMT2A* interpreted as *KMT2A* partial tandem duplication in UPNs 22, 84, 100, 123, 195 and 198.

**
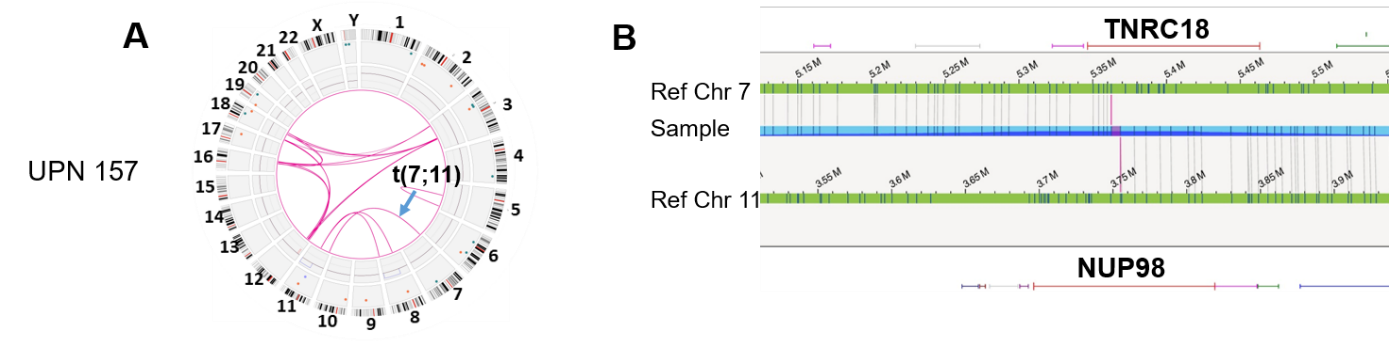
**

**Fig.S11. Example of *NUP98* rearrangement revealed by OGM (UPN 157)**

A-B) UPN 157. A) Circos plot showing a balanced translocation between chromosomes 7 and 11 (blue arrow). B) Fine mapping of the chromosomes 7 and 11 indicating a translocation t(7;11)(p22.1;p15.4)(5362596;3755020) with a breakpoint located in *TNRC18* on 7p22.1 and *NUP98* on 11p15.4.

**
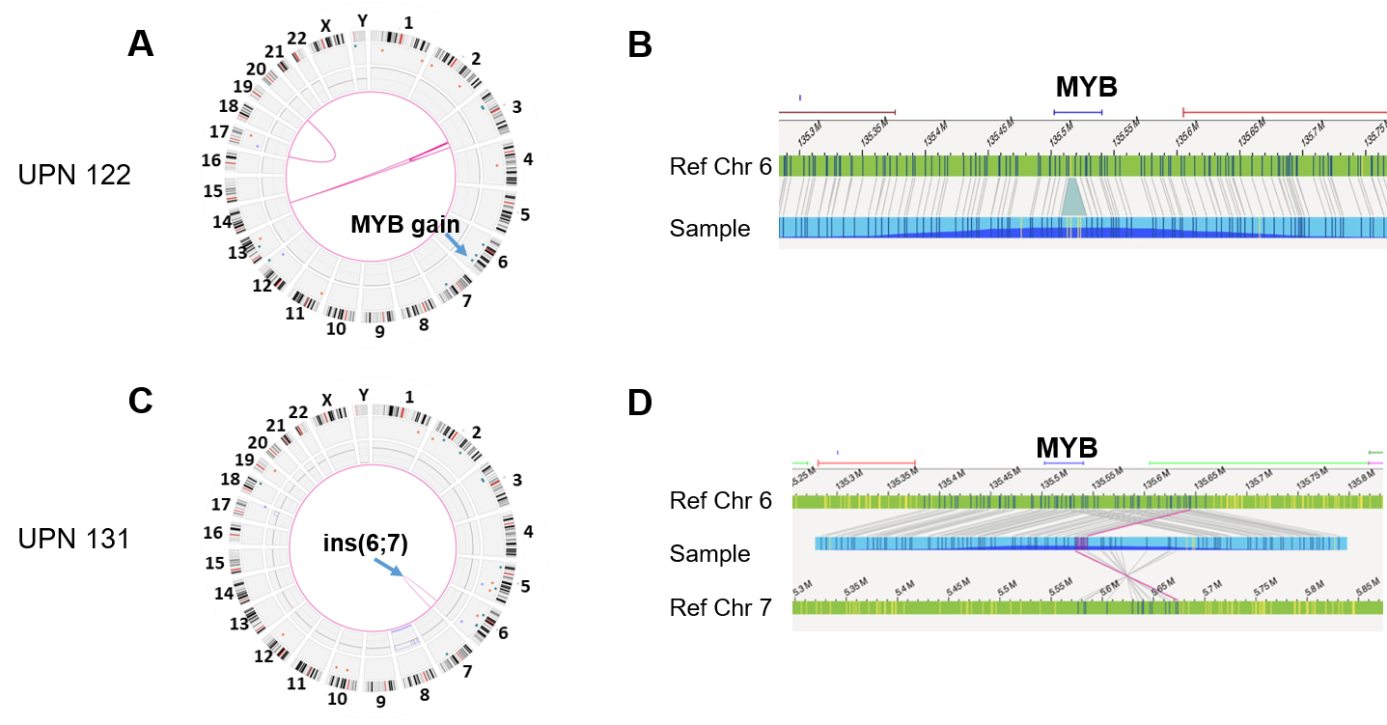
**

**Fig.S12: Examples of SVs affecting the *MYB* gene revealed by OGM (UPNs 122 and 131)**

A-B) UPN 122. A) Circos plot showing a focal SV located in the long arm of chromosome 6 (blue arrow). B) Fine mapping of the chromosome 6 indicating a focal insertion interpreted as a focal duplication in the *MYB* gene on 6q23.3. C-D) UPN 131. C) Circos plot showing an insertion from the chromosome 7 in the chromosome 6 (blue arrow). D) Fine mapping of the chromosomes 6 and 7 indicating an insertion ins(6;7)(q22.3;p22.1p22.1)(135523368;5576476_5675285) located in the *MYB* gene.


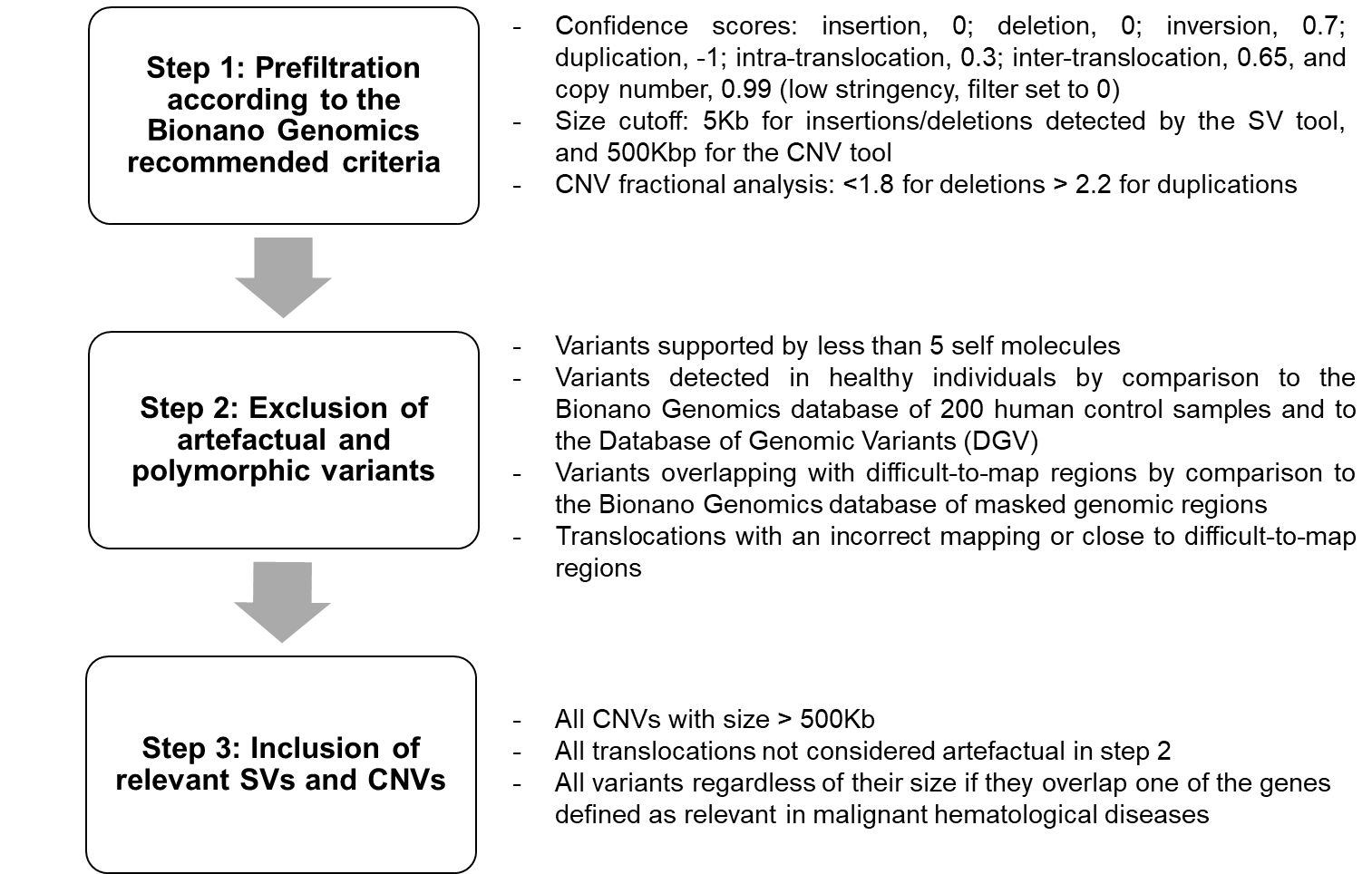


**Fig.S13. Flowchart for filtering OGM variants.**

**SUPPLEMENTAL TABLE LEGENDS**

**Table S1. Patients’ characteristics**

NA, not available

**Table S2. Patients’ routine cytogenetic and OGM results.**

* The relevant genes described here are listed in Table S7.

Cytogenetic abnormalities detected by OGM but not seen by karyotype are indicated in blue.

Abn, Abnormalities; Cx, Complex rearrangement; Cth, Chromothripsis

**Table S3. Abnormalities missed by OGM analysis**

*Cytogenetic abnormalities detected by karyotype but missed by OGM are indicated in red.

**Table S4. Prediction of risk score in MDS patients using IPSS-R risk score according to the cytogenetic method (karyotype/FISH versus OGM).**

NA, not available (because of karyotype failure)

**Table S5. Prediction of risk score in AML patients using ELN 2010/2017 risk scores according to the cytogenetic method (karyotype/FISH versus OGM).**

NA, not available (because of karyotype failure)

**Table S6. Technical performances of OGM analysis**

Avg, average

**Table S7. List of relevant genes involved in malignant hematological diseases**

**Table S8. Mutations in AML patients**NA, not available; WT, wild type; r, ratio; ITD, internal tandem duplication

Supplemental Tables are provided as separate files.
